# Mechanical splitting of microtubules into protofilament bundles by surface-bound kinesin-1

**DOI:** 10.1038/srep39408

**Published:** 2016-12-21

**Authors:** Virginia VanDelinder, Peter G. Adams, George D. Bachand

**Affiliations:** 1 Center for Integrated Nanotechnologies, Sandia National Laboratories, PO Box 5800, MS 1303, Albuquerque, NM, 87185, USA; 2 Center for Integrated Nanotechnologies, Los Alamos National Laboratory, Los Alamos, NM, 87545, USA

## Abstract

The fundamental biophysics of gliding microtubule (MT) motility by surface-tethered kinesin-1 motor proteins has been widely studied, as well as applied to capture and transport analytes in bioanalytical microdevices. In these systems, phenomena such as molecular wear and fracture into shorter MTs have been reported due the mechanical forces applied on the MT during transport. In the present work, we show that MTs can be split longitudinally into protofilament bundles (PFBs) by the work performed by surface-bound kinesin motors. We examine the properties of these PFBs using several techniques (e.g., fluorescence microscopy, SEM, AFM), and show that the PFBs continue to be mobile on the surface and display very high curvature compared to MT. Further, higher surface density of kinesin motors and shorter kinesin-surface tethers promote PFB formation, whereas modifying MT with GMPCPP or higher paclitaxel concentrations did not affect PFB formation.

Nature has evolved and optimized an extensive library of biomolecular machines, some of which have been co-opted for performing useful tasks in hybrid nano- and microscale devices and systems^1^,^2^,^3^. For instance, photosynthetic reaction centers and light-powered proton pump proteins have been used for energy harvesting^4^, while motor proteins (e.g., myosin) have been used to transport analytes and reagents in microassays^5^,^6^. In particular, the active transport system consisting of kinesin motors and microtubule (MT) filaments has been reconstituted *ex vivo* to provide transport in a range of applications including active assembly of nanocomposites, analyte separation in bio-assays, and performing molecular computation^5^,^7^,^8^. In the majority of these systems, surface-adhered kinesin-1 proteins are used to propel MTs above the surface in an inverted gliding assay format, as shown in Fig. 1A. Myriad schemes have been developed for controlled cargo binding and release, such as using DNA or antibodies to tether cargo to MTs^5^,^7^. Further, a variety of techniques have been developed to steer or change the speed or direction of MTs in a gliding assay, including microfluidic channel design, heat-activated polymers, light-controlled kinesin mutants, and applied magnetic forces^7^. One of the limitations in applying biomolecular machines, such as kinesin, in *ex vivo* applications is the breakdown of components and degradation in performance over time. *In vivo*, repair mechanisms exist to actively replace broken and/or worn-out components^9^. For instance, the D1 protein in Photosystem II must be replaced every 10–100 million cycles *in vivo* due to photodamage^10^. The lack of such repair mechanisms *ex vivo* motivates our understanding of the mechanisms underlying component breakdown and wear.

The practicality of using the kinesin/MT system to power transport in lab-on-a-chip assays depends in large part on the stability of the different components^11^,^12^. The relative stability of MTs may be related to their highly dynamic nature, which is critical to many of their *in vivo* functions. MTs are hollow filaments composed of thirteen protofilaments formed through the end-to-end polymerization of α,β-tubulin dimers^11^,^12^,^13^. The anisotropic structure of the dimer building blocks gives rise to an inherent polarity that plays a central role in directing the bidirectional transport of intracellular cargo by molecular motors such as kinesin^5^. *In vivo,* MTs display unique polymer dynamics known as dynamic instability, which is characterized by stochastic switching between states of slow growth and rapid shrinkage. The hydrolysis of the guanosine triphosphate (GTP) associated to the β-tubulin subunit plays a critical role in dynamic instability. Specifically, hydrolysis of this GTP at the plus end of the MT causes the dimer to adopt a curved conformation^14^ and curl out in a “ram’s horn” configuration. This conformation weakens the lateral interactions between protofilaments and leads to rapid depolymerization^4^,^13^,^15^,^16^.

In *ex vivo* experiments, MTs are commonly stabilized with paclitaxel (Taxol^®^), which binds specifically and stoichiometrically to β-tubulin and induces it to maintain the straight confirmation even with hydrolysis of GTP^14^,^17^,^18^. Despite the ability to stabilize their dynamics outside of the cell, MTs remain susceptible to damage during gliding motility. Breakage of MTs into two shorter MTs is commonly observed and has been attributed to photodamage or the presence of inactive kinesin motors^19^. Other distinct failure mechanisms of MTs have been shown to decrease the average length of MTs over time^20^. For example, molecular wear in which MT shortening occurs during transport was recently reported in gliding assays^20^, in which the length of MTs was shown to decrease slowly over time as the “weakest” tubulin dimers are removed molecule-by-molecule from the ends of the MT filament. The rate of molecular wear shows a complex dependence on both the surface density of kinesin and the velocity of transport^20^. In the present work, we describe a novel mechanism of MT damage in which MTs are sheared longitudinally as they are transported by kinesin motors in a gliding motility assay, leaving fragments consisting of curved protofilament bundles (PFB) reminiscent of the ram’s horn conformation. The PFBs continue to be mobile on the surface and display very high curvature compared to MTs. We further describe the dependence of this phenomenon on the surface density of the motor protein and the length and flexibility of the linker connecting the motor protein to the surface.

## Results

### Splitting of MT into PFBs

**Figure 1:**
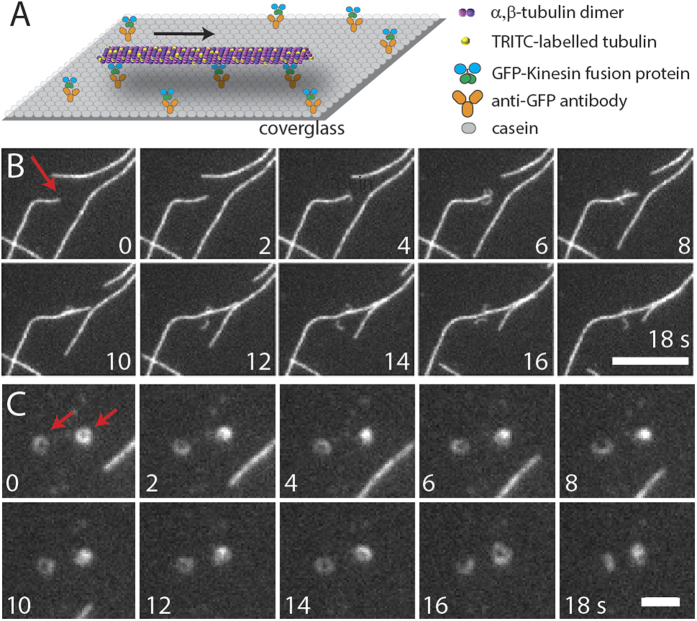
(**A)** Cartoon of motility assay system. (**B**) Fluorescence images of TRITC-labelled MT (designated by red arrow in first frame) splitting into two PFBs at 4 s that break off of the MT at 8 s and continue to move on the surface. Scale bar is 5 μm. (**C**) Fluorescence images of mobile PFBs (designated by red arrows in first frame). Scale bar is 1 μm.

MTs labelled with TRITC were observed using fluorescence microscopy in an assay where MTs glide across the surface propelled by surface-tethered kinesins, as shown in Fig. 1A. A layer of casein on the surface is used to help preserve kinesin functionality^21^. Using a GFP-kinesin-1 fusion protein adsorbed to a coverglass via an anti-GFP antibody, we observed splitting of MTs longitudinally into two fragments, which we propose are PFBs as shown in Fig. 1B. (Note that although the GFP in the GFP-kinesin fusion protein is often used in standard format assays to observe motion of the kinesin motors along immobilized MTs, in this instance the GFP is only used to attach the kinesin to the surface using anti-GFP antibodies, instead of for its fluorescence properties. Images of fluorescence from GFP-kinesin is shown in Fig. S1.) The images show static positions of the MTs; motility can be observed in the Movies (in the SI). These PFBs (Fig. 1B) break off from the parent MT approximately four seconds after the splitting begins. Analysis of the two PFBs and the parent MT suggest that fluorescence intensities of the PFBs are 48 and 45% of the parent MT. Analysis of seven splitting events show that the intensity of the daughter PFBs are 50 ± 14% (variation given is standard deviation, here and throughout the rest of this article; N = 14) of the parent MT. These data, shown in Fig. S2, suggest that the PFBs contain multiple protofilaments, but not the original number in the parent MT. The PFBs also display a higher curvature than the original MT, which is addressed in detail in the discussion section below. PFBs were also observed to split off of the tail of MTs and rarely to split off of the middle of a MT. PFBs continue to be mobile on the kinesin coated surface (Fig. 1C), and commonly form small rings with sub-micron diameters that continue to rotate over time. The PFBs are considerably less stable than the parent MTs, breaking into smaller and smaller pieces with a half-life on the order of 30 ± 20 s (N = 5, as measured by the fluorescence intensity from the time a PFB splits off from a MT). Additional splitting events are shown in the Supplementary Information (Movies S1,S2,S3).

### Velocity of MTs and PFBs

The velocity of the “parent” MTs on the surface is consistent with that reported for full-length *Drosophila* kinesin-1 adhered to the surface through adsorption, 480 ± 60 nm/s (N = 133)^19^. However, the PFBs displayed a considerably lower velocity, 270 ± 110 nm/s (N = 76); MT motion and PFB formation was not observed in the absence of ATP (i.e., 0 mM ATP).

### SEM and AFM Imaging

**Figure 2:**
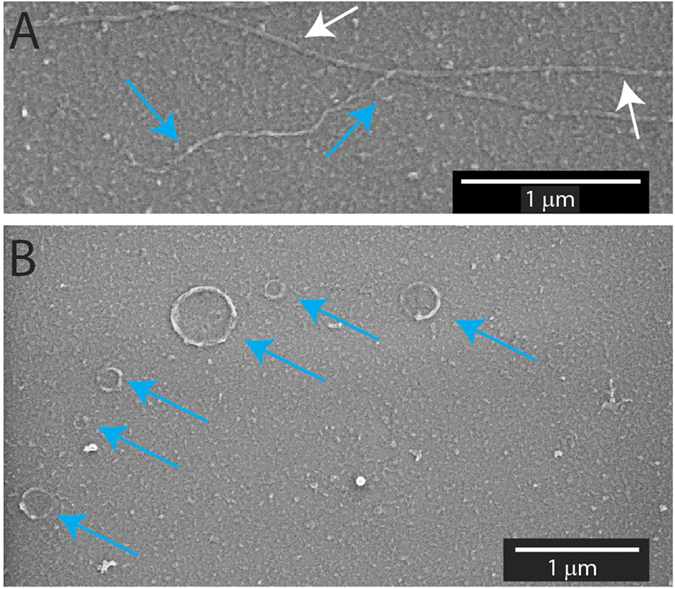
High resolution SEM images of MTs and PFBs (highlighted by white and blue arrows, respectively). (**A**) Image of an intact MT and an overlapping MT whose end is splitting into PFBs. (**B**) PFBs displaying small radius of curvature.

**Figure 3:**
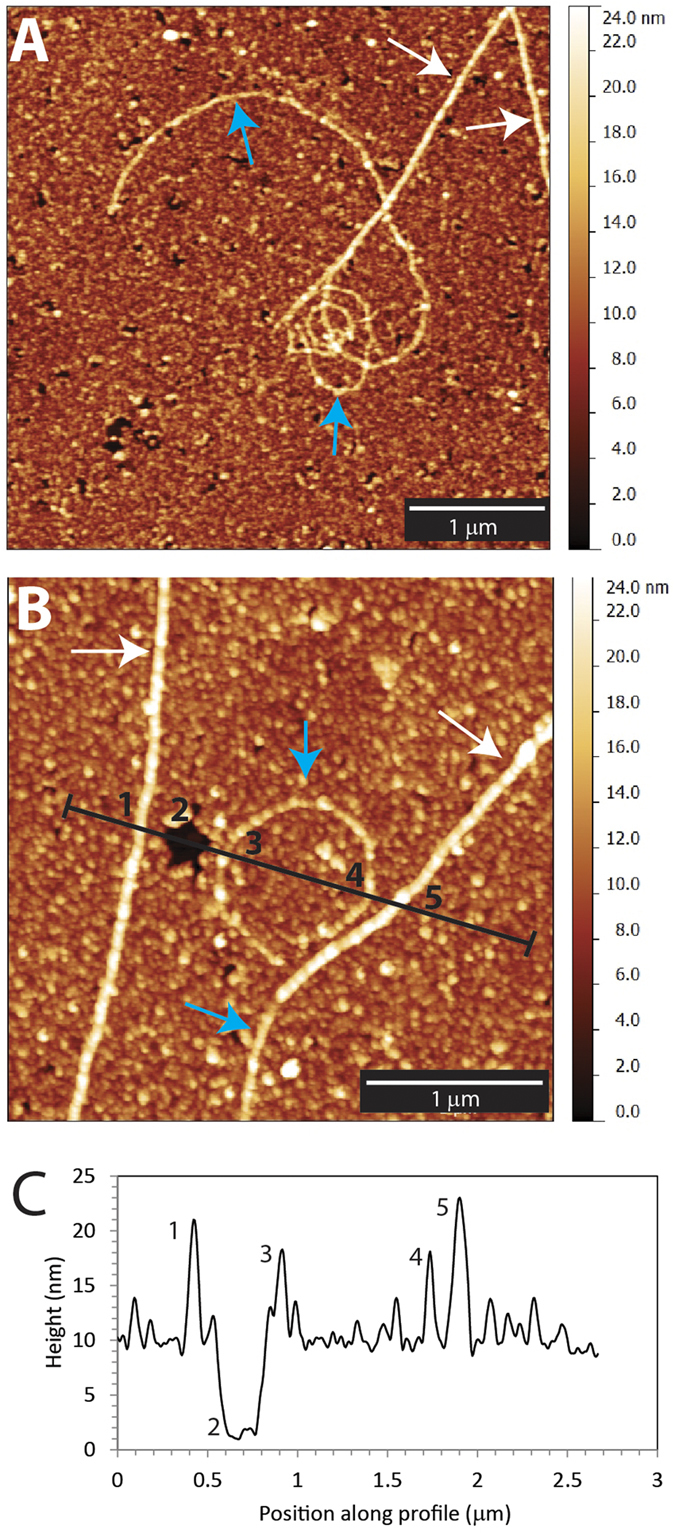
High resolution AFM images of MTs and PFBs (highlighted by white and blue arrows, respectively). (**A**) Image of MT splitting to PFBs. (**B**) Image of a MT, curved PFB, and a MT with a PFB still attached to the end. (**C**) Example height profile along black line shown in (**B**), with corresponding MT and PFB peaks and glass surface are labeled in both (**B**) and (**C**).

Due to the resolution limits of fluorescence microscopy, we also characterized the PFBs with scanning electron microscopy (SEM) and atomic force microscopy (AFM). By preparing the samples for SEM or AFM (drying and fixation), one may assume that MTs are effectively frozen in their current state, so we sample a population of intact and split MTs. Representative SEM and AFM images of the MTs and PFBs are shown in Figs 2 and 3, respectively; a gallery of SEM images may be found in Fig. S3. An intact MT and a MT whose end has split into two PFBs are shown in Fig. 2A. Here, one of the PFBs is relatively short while the other is substantially longer and winding. The thickness of the parent MT is ~30 nm, while the thickness of the long and short PFBs are ~20 nm and ~10 nm, respectively. The diameter of a MT composed of thirteen protofilaments is approximately 25 nm^5^; sample preparation for SEM is likely to account for the discrepancy between the measured and expected values. Figure 2B shows six PFBs that have curled up into submicron rings. The average inner and outer diameter of submicron rings observed by SEM was 210 ± 150 nm and 280 ± 170 nm, respectively (N = 14). The resultant ring thickness of ~35 nm suggests that the rings on average consist of more than one loop of PFBs.

Figure 3A and B show AFM images of a MT splitting into two PFBs where one of the PFBs has broken off from the MT while the other remains attached. In both cases, the PFBs display a much greater curvature than the microtubule. A gallery of AFM images showing PFBs and rings can be found in Fig. S4. Height profiles derived from these images (Fig. 3C) suggest that the casein protein layer was 10 ± 0.6 nm (mean ± standard deviation) thick. The height of MTs was 12 ± 1 nm above the casein layer while PFBs had an average height of 6.8 ± 1.4 nm above the casein layer. As the AFM was performed on dried samples, the heights are expected to reflect relative differences between intact MTs and PFBs, and be smaller than the actual sizes previously reported (shrinkage due to desiccation has often been reported in previous AFM studies on other materials).

### Curvature of PFBs

The curvature of the PFBs and MT in the SEM and AFM images was determined by either fitting a circle to the fragment to obtain the radius of curvature or by using the method of Bicek *et al*.^22^. Briefly, the length of the MT or fragment was fitted with a curve. The curvature, *K*, was then determined by averaging four points together to smooth the curve and then computing the change in angle ∅_*k*_ between three adjacent points and dividing by the average arclength of the two adjacent segments, 
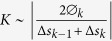
. The radius of curvature is the inverse of the curvature, 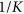. The average radius of curvature of the PFBs was calculated as 0.23 ± 0.2 μm (N = 40), while the average radius of curvature of MTs was approximately 100-fold greater (20 ± 23 μm, N = 16). Additionally, in three images PFBs were observed to be attached to the end of a MT. The average radius of curvature of these end-attached PFBs was 2.5 ± 1.9 μm (N = 3). Fluorescence images show that a PFB on the tip of a MT can lead the whole MT following the curve of the tip (Fig. S5). Histograms showing the average radius of curvature of PFBs measured with SEM and AFM are shown in Fig. S6. No correlation between the radius of curvature of a PFB and its height (as measured by AFM) was observed (Fig. S7).

### Effect of surface density of kinesin

**Figure 4:**
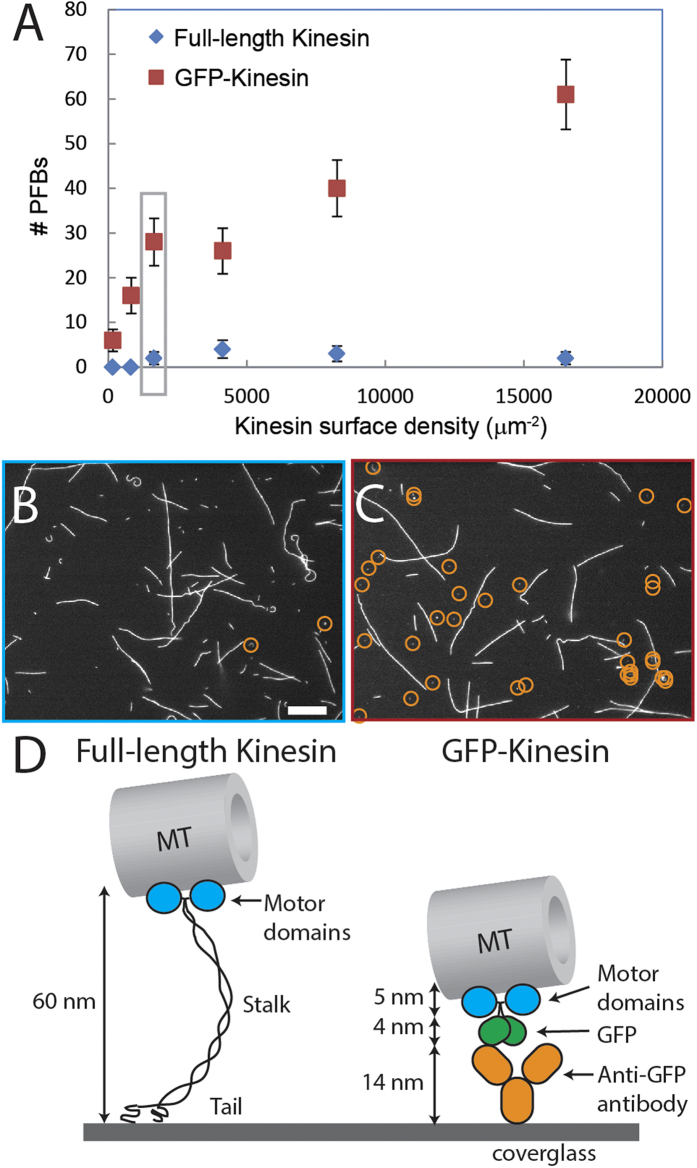
(**A**) Graph of the number of PFBs versus the kinesin surface density. Error bars are standard deviation. Fluorescence images showing PFBs (circled in orange) for GFP-kinesin (**C**) and full-length kinesin (**B**) at a kinesin surface density of 1650 μm^−2^ (data points highlighted by grey box in (**A**)). Scale bar is 10 μm. (**D**) Cartoon of the full-length and GFP- kinesin constructs and attachment schemes to surface.

The rate of PFB formation depended on the surface density of kinesin. Following the procedure of Hancock and Howard^23^, we calculated the surface density of kinesin using the total protein concentration and the dimensions of the flow channels experiments were performed in. The amount of antibody used to attach the GFP-kinesin was always in molar excess of the amount of GFP-kinesin, while the GFP-kinesin concentration was varied to change the surface density. We verified that the surface density of two different motor constructs was the same at a given protein concentration (0.36 μM) using MT landing rate measurements (landing rates *R*_*full-length*_and *R*_*GFP-kinesin*_ are 21 ± 8 s^−1^ and 22 ± 7 s^−1^, respectively; see Methods section and Fig. S8 for details). The main uncertainty in these calculations is the fraction of inactive motors, which can be as high as 50% and can be different for the two motor constructs^23^. Figure 4A–C and S1 show a positive correlation between the surface density of kinesin and the number of PFBs formed. Specifically, more PFBs are formed at high kinesin surface density, whereas very few PFBs are formed at low kinesin surface density. The data presented in Fig. 4A–C and S1 are a result of counting the number of PFBs for each condition at the same time point (30 min after wash with motility solution). The half-life of the PFBs was similar for both the full-length and the GFP-kinesin (90 ± 70 s and 50 ± 40 s, respectively, from measurements of decay in fluorescence of PFBs), indicating that the difference in PFB number in the two cases is due to differences in the rate of PFB formation, rather than their longevity. The surface density experiments were repeated separately an additional two times using the same concentration of microtubules with the same initial length distribution; images were recorded at each condition at the same time point were recorded for each experiment. While the exact number of PFBs formed under each condition varied, the data from the replicate experiments confirmed the positive correlation between motor surface density and formation of PFBs.

### Effect of kinesin linker length/flexibility

The flexibility and length of the linker between the kinesin motor domains and the surface also affect the formation of PFBs. Cartoons of two kinesin constructs used in these experiments are shown in Fig. 4D. Full-length kinesin-1 (from *Drosophila*) has a long, flexible linker consisting of alpha helices and hinged domains connecting the motor domain to the tail region, which attaches to the surface. As shown by Dumont *et al*.^24^, the height of the head domain away from the surface depends on the kinesin surface density, with the linker adopting either a mushroom configuration at low densities or an extended brush configuration at high densities, with the height of the MT from the surface varying between 15 and 60 nm, respectively^24^. In the GFP-kinesin fusion protein, the kinesin has been truncated to the first 430 amino acids, which includes only the motor domain and the neck region, but not the long linker domain. The neck region of the kinesin is attached to a GFP protein, which is barrel-shaped, approximately 4.2-nm long and 2.4 nm in diameter. The protein is attached to the surface via an anti-GFP antibody, which is ~14 nm in length. Therefore, the linker between the motor domains on the GFP-kinesin fusion protein is much shorter and lacks the flexible region of the full-length kinesin. As shown in Fig. 4A, the full-length kinesin produced very few PFBs compared to the GFP-kinesin fusion construct at the same kinesin surface density. Figure 4B,C shows images of one of the kinesin surface concentrations for both full-length and GFP-kinesin proteins with PFBs circled in yellow (images for the other concentrations are shown in Fig. S1). Due to the unknown fraction of inactive motors, it is possible that the surface density of active GFP-kinesin motors is higher than that of the full-length kinesin at the same nominal concentration. The occurrence of more pinned MTs suggests that the fraction of inactive full-length kinesin is likely greater than that of the GFP-kinesin motors. While the surface density is an important factor in the formation of PFBs, the considerable difference in PFB formation between the two kinesin constructs is too large to be caused solely by different densities. Here, the nature of the kinesin surface tether likely has a significant role in the formation of PFBs, and is an area for future study.

### Effects of MT composition

The effect of changing the experimental buffers and MT properties on the formation and longevity of PFBs was investigated. In these experiments, the assay was run for 30 minutes and then the number of PFBs and MTs was measured. We first performed experiments with three different paclitaxel concentrations: 1, 10, and 50 μM (with 10 μM being the value used in all other experiments). PFB formation was observed for all concentrations of paclitaxel with no detected changes in morphology by fluorescence microscopy (Fig. S9A–C). However, in comparison to the 10 μM condition, PFBs were less and more stable at 1 and 50 μM paclitaxel, respectively (Fig. S9D). To examine the potential role of tubulin modification and photodamage, MTs were prepared with two different percentages of TRITC-labelled tubulin. As shown in Fig. S10, no differences in PFB formation or morphology were observed. Because different MT samples were used, the number of PFBs was corrected for the number of MTs, with 0.8 ± 0.3 and 0.8 ± 0.2 PFBs per MT per field of view for the 20% and 50% TRITC-labelled tubulin MTs, respectively. In experiments using “double stabilized” MT (i.e., paclitaxel plus GMP-CPP, a non-hydrolysable analog of GTP), an increase in the stability PFBs was observed compared to single stabilized MTs, as shown in Fig. S11. Because different MT samples were used, the number of PFBs was corrected for the number of MTs, with 2.5 ± 0.2 and 1.6 ± 0.3 PFBs per MT per field of view for the GMP-CPP and GTP MTs, respectively. No changes in the morphology of PFBs were evident via fluorescence microscopy.

## Discussion

**Figure 5:**
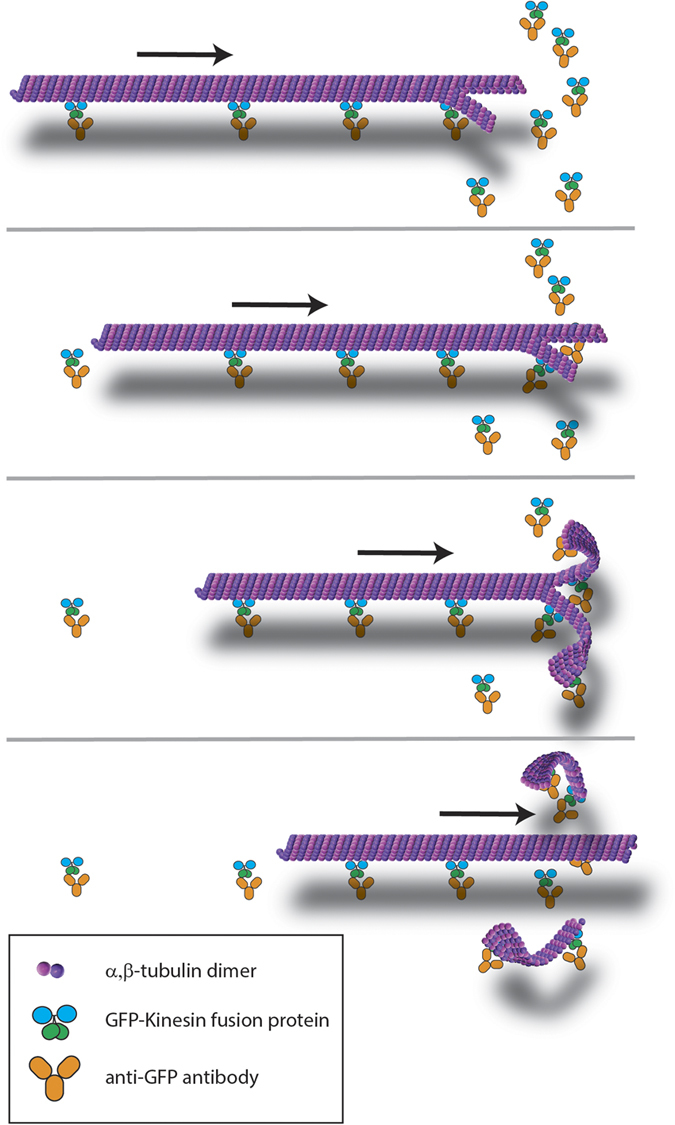
Cartoon of proposed mechanism of MT splitting into PFBs by GFP-kinesin. A MT with PFB tails on the leading end encounters kinesin motor at the correct configuration so that the motors each bind to one of the PFBs. The kinesin motors then unzip the MT into the corresponding PFBs, which break off of the parent MT.

We have described a new mechanism of MT breakage in which instead of shearing into two shorter MTs, the MT splits longitudinally into two PFBs (Fig. 5). MTs, even those stabilized with paclitaxel, have slightly frayed ends, or tails^25^. Splitting is initiated when a MT with a frayed end on the leading end encounters kinesin motors in a configuration such that separate motors bind to different parts of the end. As they pass these motors, the MT unzips longitudinally into two PFBs, each comprising about half the protofilaments of the original MT. The daughter PFBs often break off of the parent MT, which may be attributed to the observed differential velocities of the MT and PFBs. A cartoon of the proposed mechanism is shown in Fig. 5. As this process is seen to repeat multiple times (see Movies S1 and S2), the ends that are left behind when PFBs split off of MTs must be frayed as well. As shown in the image sequences in Fig. S2, the point where splitting occurs is fixed, which is consistent with the proposed model.

It has previously been reported that a small percentage of MTs prepared in the presence of stabilizing agents, such as paclitaxel, DMSO, and glycerol, possess a short, coiled structure on their end, dubbed “tails”^26^,^27^,^28^. Based on the relative brightness and shape of the tails, Ray *et al*. concluded that the tails were incomplete MTs containing fewer than eight protofilaments with a C-shaped cross section, rather than the normal, closed O-shaped configuration^29^. We hypothesize that these tails are the precursors to the formation of PFBs described in the present work. Specifically, our data suggest that the fragments breaking off of the MT during transport consist of bundles of about 5–8 protofilaments, as supported by the analysis of the fluorescence intensity, and measurement from SEM and AFM images. Each PFB has approximately half the intensity of an intact MT (Fig. 1), which agrees with measurements from SEM and AFM suggesting the PFBs are approximately half the thickness of the parent MT (Figs 2 and 3).

Another piece of evidence that the fragments do indeed consist of PFBs is their high curvature (see Fig. 2). As measured in the SEM and AFM images, the PFBs have a radius of curvature of 0.23 ± 0.2 μm as compared to 20 ± 23 μm for MTs. The closed tubule structure of MTs imparts their rigidity. Cryo-TEM studies have shown that individual GDP protofilaments have a very low radius of curvature of 0.021 ± 0.004 μm^18^. Paclitaxel stabilized protofilaments, on the other hand, have a reported radius of curvature of 0.245 ± 0.105 μm^18^, which is similar to that of the PFBs found in our work.

The motility of the fragments on the surface, as shown in the movies in the SI, is further support that the fragments consist of PFBs. In order for the fragments to be transported by the surface-bound kinesin, the binding region of the protofilament must continue to be accessible to the kinesin. The vast majority of *in vitro* research has been performed on the classic microtubule structure of tubulin. Other tubulin structures, such as zinc sheets in which protofilaments are arranged antiparallel, and C-tubules in which are oriented protofilament sheets have also been investigated^12^,^28^,^30^. Prior to this demonstration of microtubule fragments consisting of PFBs, the closest analog observed was the C-tubules studied by Kamikura *et al*.^12^. The C-tubules were formed by polymerization in the presence of DMSO, and were found to be motile on a kinesin-coated surface at a level of approximately 50%. The velocity and curvature of trajectories of the motile C-tubules was similar to normal microtubules^12^. In contrast, we observed all MT fragments to be motile with a velocity significantly lower than that of whole MTs (270 ± 110 vs 480 ± 60 nm/s). The velocity mismatch between the intact MTs and the PFBs likely causes the PFBs break off of the MTs during formation.

The stall force (*F*_*S*_) of kinesin-1 has been measured by single motor optical trapping to be 5.4 ± 1.0 pN, resulting in ~4 × 10^−20^ J of work per each step of 8 nm along a MT^31^. With one motor attached to each protofilament tail of a MT, as shown in Fig. 5, the force exerted on the last lateral tubulin-tubulin bond can be estimated by 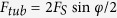, where φ is the angle between the tails. The kinesin motors will generate a maximum force when φ is 180°, or in other words when the kinesin are pulling directly against each other on the ends. Here the work done by the two kinesin motors will be ~8 × 10^−20^ J, which needs to be sufficient to break two lateral tubulin-tubulin bonds (the tails are C-shaped and connected on the top and bottom of the C’s in the MT), or ~4 × 10^−20^ J of work to split apart a single lateral tubulin-tubulin bond. This value is in reasonable agreement with that determined from *in silico* modeling of 4.8 × 10^−20^ J (6.9 ± 0.4 kcal/mol) for this bond^32^, suggesting that having one motor on each end might be sufficient to split the MT. At high motor surface densities, there are likely more than one motor on each end, which would produce ample force to split the MT. In contrast, the longitudinal tubulin-tubulin bonds, which hold the tubulin in protofilaments, are much stronger (14.9 ± 1.5 kcal/mol)^32^, explaining the separation of the MTs into PFBs.

The effect of the kinesin surface density and kinesin linker length/flexibility supports the proposed mechanism of MT splitting. As shown in Fig. 4, the rate of PFB formation depends strongly on the surface density of kinesin, with more PFBs being broken off of MTs at higher densities. Here, the formation of PFBs during transport depends on the kinesin being positioned sufficiently close to each other on the surface to permit the attachment of multiple motors to the leading ends simultaneously. In addition to the surface density, our data suggest that the length and flexibility of the tail also play a critical role in this process. Although possible differences in kinesin surface density may account for some of the decreased PFB formation with full-length kinesin, the stark difference in PFB for the two constructs indicates that the length and flexibility of the tail also play a role. Very few PFBs were observed with the full-length kinesin, even at high surface density. The rarity of PFB formation when using full-length kinesin may account for why this phenomenon has not previously been reported. Kinesin constructs with short linkers have been primarily used in the natural motility geometry in which kinesin motors walk along stationary microtubules; truncated kinesin have been used much less frequently the inverted gliding geometry. The long, flexible linker of the full-length kinesin might more easily be able to conform to the path of the MT and be unable to provide the force necessary to cleave the lateral tubulin-tubulin bonds.

Although the splitting of MTs into PFBs has not been previously reported, we believe PFBs have been observed ~25 years ago and were labeled a mysterious phenomenon: MTs in a gliding assay that traveled in arcs or formed small (several micron diameter) rings that persisted for a time before straightening out^33^,^34^. It has recently been proposed by Ziebert *et al*. that these arcs are caused by a conformational change in tubulin rearrangement of the tubulin lattice in the MT to a curved configuration^35^. However, our work offers an alternative explanation: the arcs might be caused by PFBs at the leading end of the MT. As shown in Movie S4, the MT is led in a curved trajectory by the curved PFB at leading end, until the sheet breaks off, and the MT then straightens out. Even a short leading PFB can cause curvature, and yet be too short to resolve with fluorescence microscopy, which might have led to this phenomenon being overlooked previously.

## Conclusions

In summary, we find that a high surface density of GFP-kinesin motor proteins is capable of splitting MTs into PFBs during transport in the gliding motility assay. Further studies of the system using non-processive, single-headed motors should provide a deeper understanding of this splitting phenomenon. Other MT motors, such as dynein or minus-end kinesin, as well as motors with different force generation would also provide valuable insights. We show that paclitaxel can, at least for a short time, stabilize PFBs, and that surface bound kinesin can transport these sheets across the surface, opening the door to exploring kinesin transport with novel configurations of tubulin beyond the standard MT. The results of this study demonstrate another possible mechanism of MT damage that is especially relevant in situations with a high kinesin surface density or kinesin motors with short, inflexible linkers. In order to increase fidelity of optimized gliding assays, it is important to understand these process deficiencies in order to minimize MT damage, informing future device design.

## Materials and Methods

The plasmid rkin340GFP for GFP-kinesin expression was transformed into BL21 cells, expressed, and purified following standard protocols for *E. coli* expression and purification of a His-tagged protein. In the GFP-kinesin fusion protein, the kinesin has been truncated to the first 430 amino acids, which includes only the motor domain and the neck region, but not the long linker domain, and the neck region is attached to a GFP protein. Similarly, the expression and purification of full-length kinesin (plasmid ppK113) has been described extensively^36^. The resultant protein concentrations were determined using UV-Vis absorbance measurements. The absorbance of GFP-kinesin was measured at the GFP absorbance peak of 488 nm, and the extinction coefficient for GFP, 56,000 M^−1 ^cm^−1^, was used to calculate the concentration. The absorbance of the full-length kinesin was measured at 280 nm, and the extinction coefficient of 42,500 M^−1 ^cm^−1^ was calculated based on the protein sequence.

TRITC-labeled MTs were made by polymerizing TRITC-labeled tubulin and unlabeled tubulin (20 to 80 molar ratio, respectively (unless stated otherwise); 1.3–1.65 mg/mL final concentration; Cytoskeleton, Inc.) in GPEM polymerization buffer (80 mM PIPES, 4 mM MgCl_2_, 1 mM EGTA, 1 mM GTP; pH adjusted to 6.9 with KOH; Sigma). MTs were polymerized at 37 °C for 30 min and then stabilized in BRB80T (BRB80: 80 mM PIPES, 1 mM MgCl_2_, 1 mM EGTA; 10 μM paclitaxel; pH adjusted to 6.9 with KOH) at a final concentration of 13–16.5 μg/mL. GMPCPP MTs were made using the same protocol except substituting 1 mM GMPCPP for the GTP in the polymerization buffer.

Glass flow cells were made of a glass slide and coverslip connected by two pieces of double-sided sticky tape resulting in a 3 by 22 mm channel. For assays with full-length kinesin, 20 μL of kinesin solution in BRB80 with 10 mM ATP and 0.2 mg/mL casein (BRB80CA) was introduced to the flow cell and allowed to incubate for 5 min. Then 20 μL of MT in motility solution, which consisted of 1.3 μg/mL tubulin in motility solution with an oxygen scavenger system (0.2 mg/mL casein, 1 mM ATP, 0.02 mg/mL glucose oxidase, 0.008 mg/mL catalase, 20 mM D-glucose, 1 mM DTT, and 1 mM trolox in BRB80T) was incubated in the channel for 5 min. (Following the protocol of Cordes *et al*., the solution was exposed to 302 nm light to convert 100 μM of the trolox (TX) to the quinone (TQ) form. The concentration of TX and TQ as a function of UV light exposure time was monitored using the UV absorbance spectroscopy)^37^. Finally, the flow cell was washed with 20 μL of motility solution before imaging. For experiments with GFP-kinesin, 0.4 mg/ml anti-GFP antibodies (Abcam) in BRB80 were introduced to a glass flow cell and allowed to incubate for 5 min. Next, the channel was washed with 20 μL of GFP-kinesin in BRB80CA and let incubate for 5 min. Then 20 μL of MTs in motility solution were added and let incubate for 5 minutes. Finally, the flow cell was washed with 20 μL of motility solution before imaging. The assays were imaged with an Olympus IX71 microscope with a 100 × 1.4NA oil immersion objective, Orca3CCD camera (Hamamatsu), ND25 neutral density filter, and a TRITC filter set.

To determine relative kinesin surface densities, landing rates of MTs were measured following the protocol in Lam *et al*.^38^. The procedures listed above were followed for two flow cells: one with full-length kinesin, and the other with GFP-kinesin, both at the same purported concentration (0.36 μM). However, all ATP in the solutions was substituted for AMP-PNP, a non-hydrolysable analog, to prevent kinesin-powered gliding of MTs. Also, the MTs were passed through a 27 gauge needle 5 times to shorten them. The flow cells were sealed to prevent evaporation. The number of MT in a field of view was recorded as a function of time every 4 minutes for 20 minutes. The landing rate, *R*, was obtained using 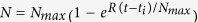, where *N* is the number of MTs in the field of view, *t* is the time, and *t*_*i*_ is the initial time MTs were introduced into the flow cell. *R* was determined for each set of time points and then averaged to obtain a landing rate for each kinesin construct.

Image analysis was performed using ImageJ. Fluorescence intensity was measured by taking a profile of intensity perpendicular to the MT or PFB averaging over at least 5 pixels. PFBs were distinguished from MTs in videos using both fluorescence intensity and the type of motion exhibited: MTs follow almost straight trajectories while PFB trajectories display a large amount of curvature.

AFM was performed using an MFP-3D-SA system, equipped with a closed loop XY scanner and all-digital ARC2 Controller (Asylum Research, Santa Barbara, CA). Samples were prepared as described above and then fixed using 1–1.5% glutaraldehyde for 30 minutes, washed with DI water, and then dried. Imaging was performed in AC mode, in air, using Olympus AC240TS Si probes (k ~2 N/m). High quality topographs were generally acquired at 512 × 512 pixels, 1 Hz scan speed, with other parameters optimized while scanning to impart minimal forces. Images were processed and height profiles generated using Gwyddion software (v2.38, open source). Height profiles of PFBs and MTs were made by averaging together either five or ten points together to make a profile perpendicular to the MT, and this measurement was repeated at multiple places along each MT or PFB. Six MTs and 20 PFBs were used in the height and curvature analysis.

SEM samples were prepared following the same protocol as AFM samples. Then a thin layer of gold was sputtered onto the dried samples. The analysis was performed using a Zeiss Supra 55VP, field emission gun scanning electron microscope. Analysis of images was performed with ImageJ.

## Additional Information

**How to cite this article:** VanDelinder, V. *et al*. Mechanical splitting of microtubules into protofilament bundles by surface-bound kinesin-1. *Sci. Rep.* **6**, 39408; doi: 10.1038/srep39408 (2016).

**Publisher's note:** Springer Nature remains neutral with regard to jurisdictional claims in published maps and institutional affiliations.

## Supplementary Material

Supplementary Movie S1

Supplementary Movie S2

Supplementary Movie S3

Supplementary Movie S4

Supplementary Information
