# Supplementary material for: Mechanical splitting of microtubules into protofilament bundles by surface-bound kinesin-1
Source: Sci Rep. 2016 Dec 21;6:39408. doi: 10.1038/srep39408 (PMC5175155; doi:10.1038/srep39408)
Supplement: Supplementary Information [file srep39408-s5.pdf]

Mechanical splitting of microtubules into protofilament bundles by surface-bound kinesin-1

Virginia VanDelinder, Peter Adams, George D. Bachand

SUPPLEMENTARY INFORMATION

Supplementary Figure 1

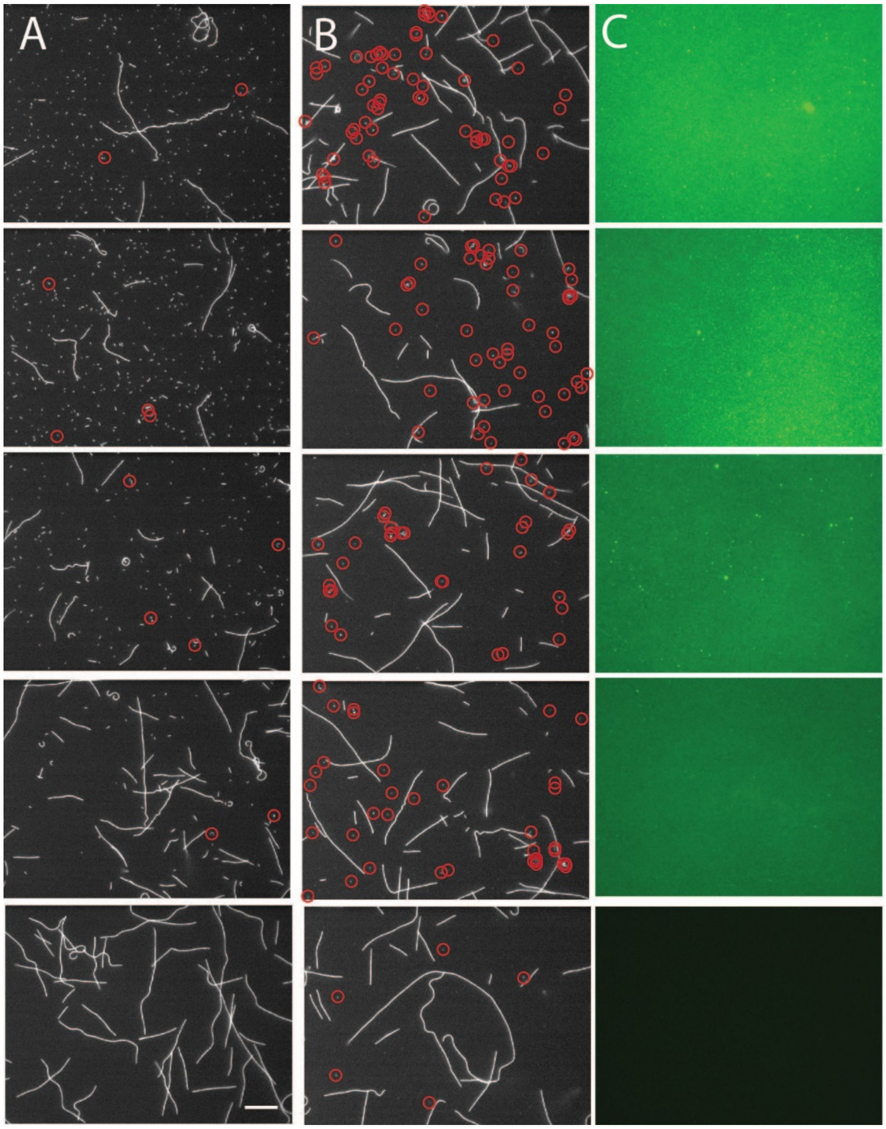

Figure S1. Fluorescence images with fragments circled in red for full-length kinesin (A) and GFP-kinesin (B) both with a TRITC-labelled MTs and a TRITC filter set, and (C) GFP-kinesin and a GFP filter set at the following concentrations from top to bottom: 16500, 8260, 4130, 1650, 165 μm^{-2}. The circled fragments were determined in the movies that the fluorescent images in (A) and (B) were taken from. Scale bar is 10 μm.

Supplementary Figure 2

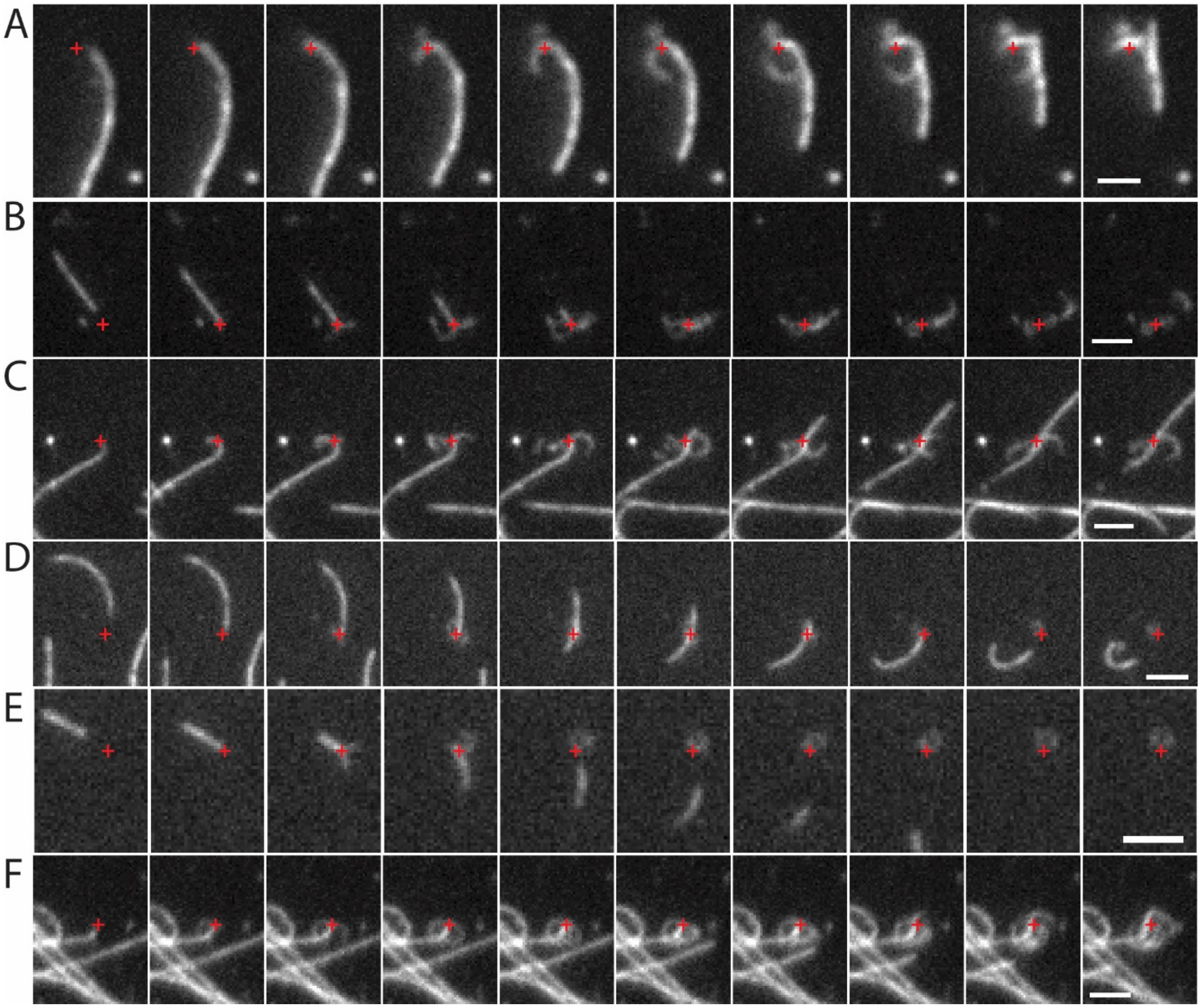

Figure S2. (A-F) Image sequences of PFB formation. Images are every 2 s. Point where splitting occurs is marked with a red "+", which is in the same location in each frame. Scale bars are 2 μm.

Supplementary Figure 3

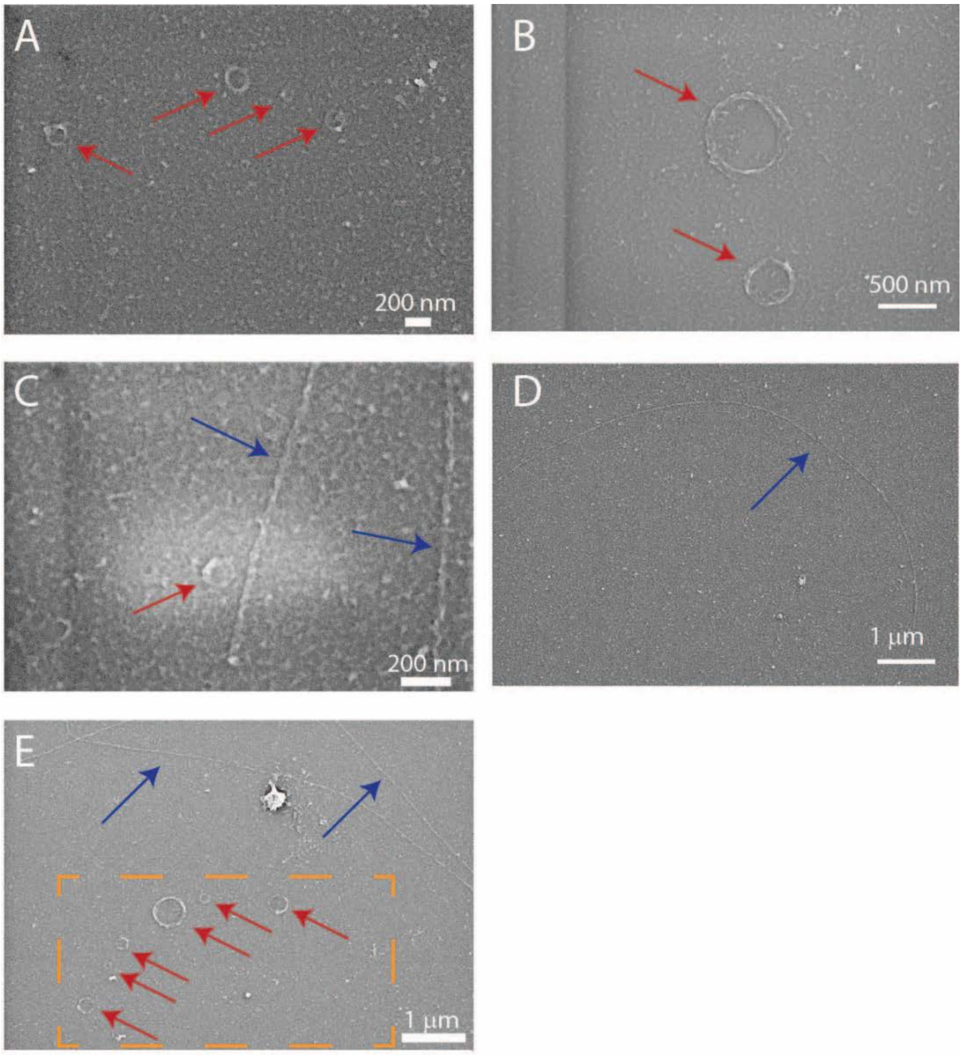

Figure S3. SEM images of MTs and fragments (highlighted by blue and red arrows, respectively).The dashed box in (E) is the region shown in Figure 2B.

Supplementary Figure 4

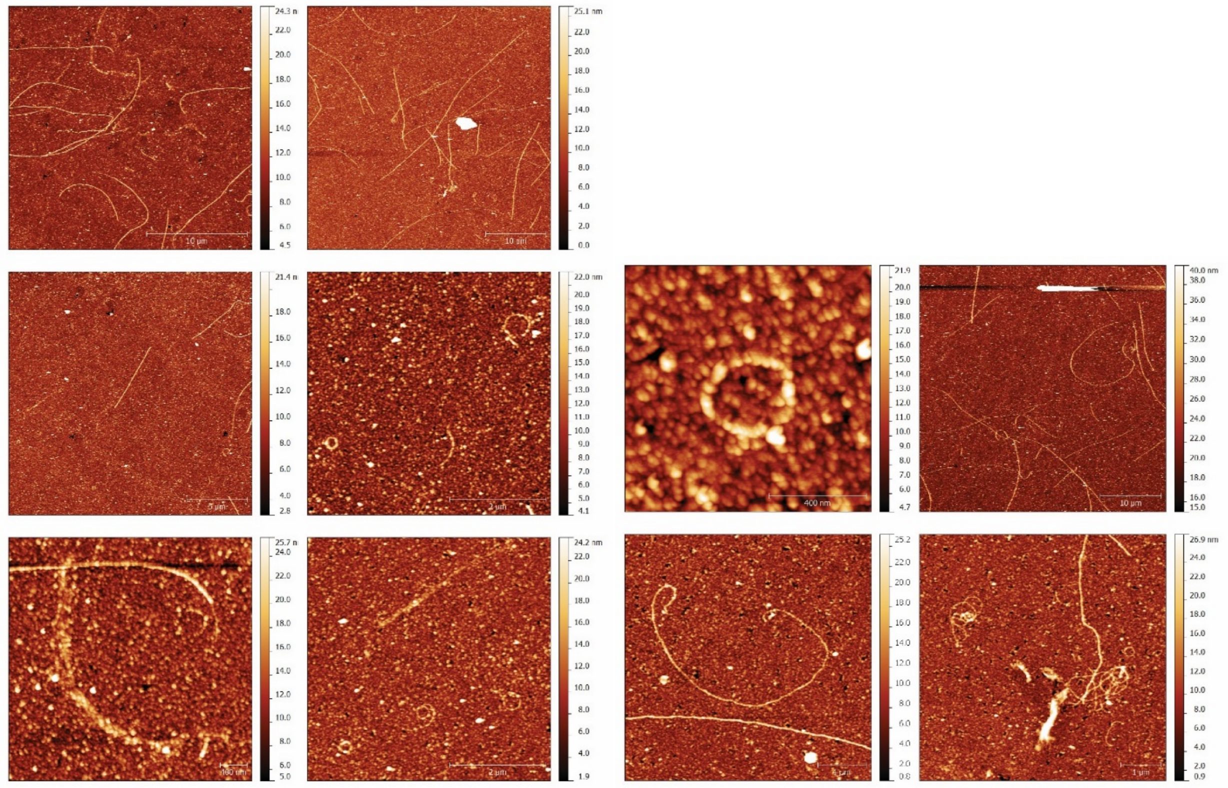

Figure S4. AFM images of MTs and PFBs.

Supplementary Figure 5

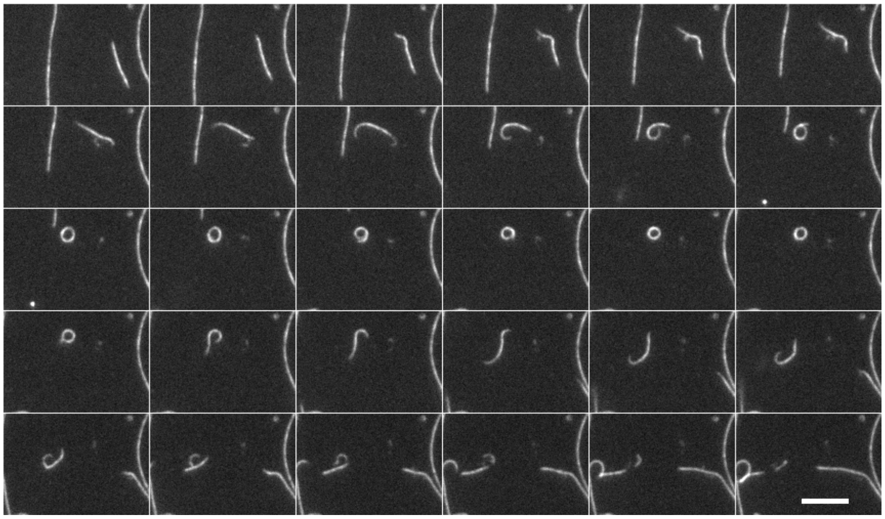

Figure S5. Fluorescence images of MT that had a fragment split off of the leading end in the 4th frame. The curved fragment that remains attached causes the MT to follow a circular trajectory. When the fragment breaks off the MT tip, the MT again travels in a normal, straight manner. Scale bar is 5 μm; 2 s between each frame.

Supplementary Figure 6

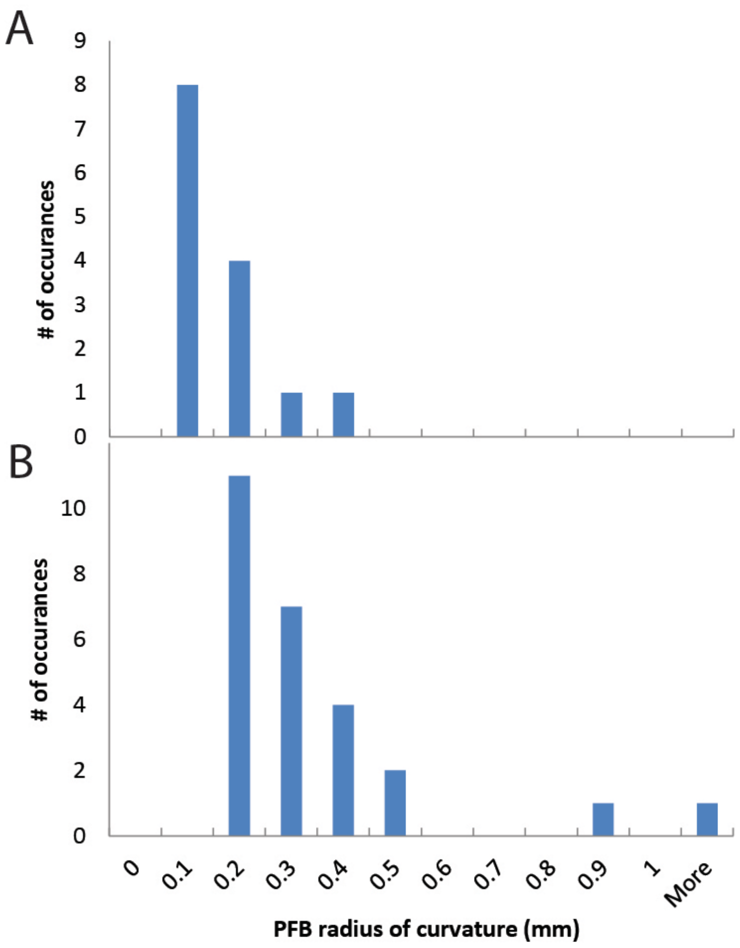

Figure S6. Histograms of radius of curvature of PFBs as measured with SEM (A) and AFM (B).

Supplementary Figure 7

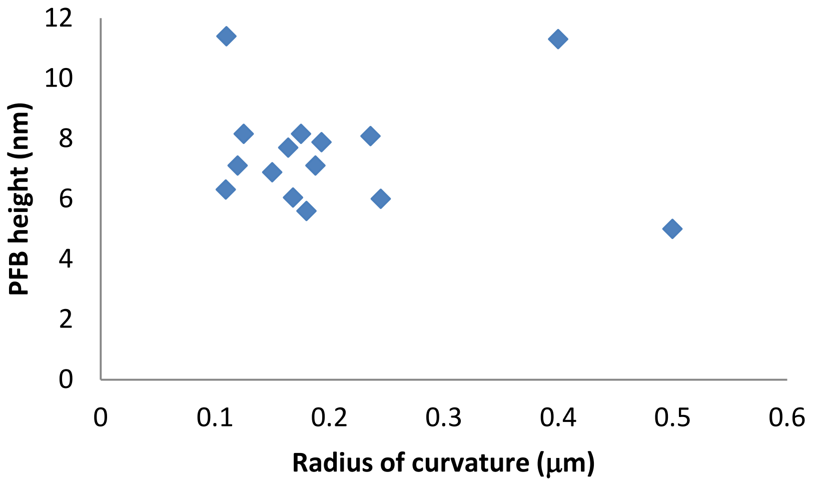

Figure S7. Plot of PFB height as measured with AFM versus PFB radius of curvature. No correlation between height and curvature was observed.

Supplementary Figure 8

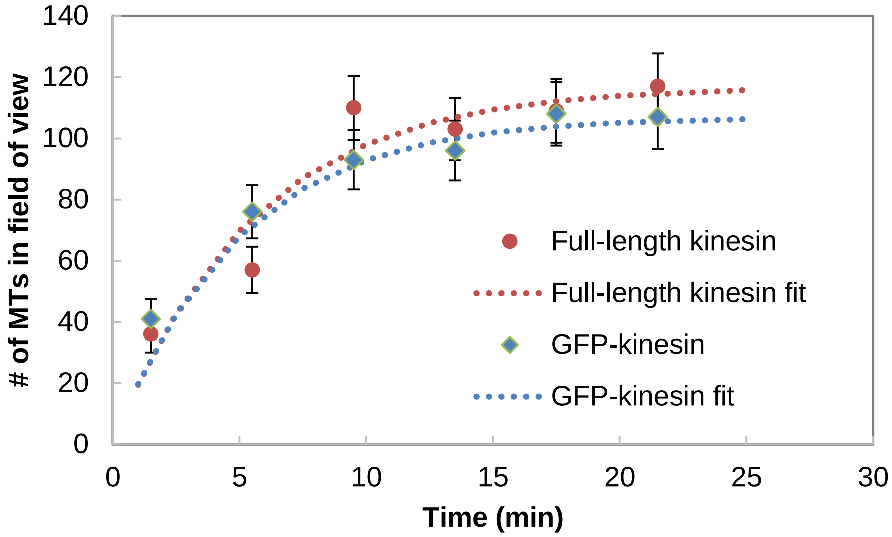

Figure S8. MT landing rates and fits for GFP-kinesin and full-length kinesin, both introduced to a flow cell at 0.36 μM concentration. Detailed description of the procedure for these measurements is provided in the Methods section of the paper.

Supplementary Figure 9

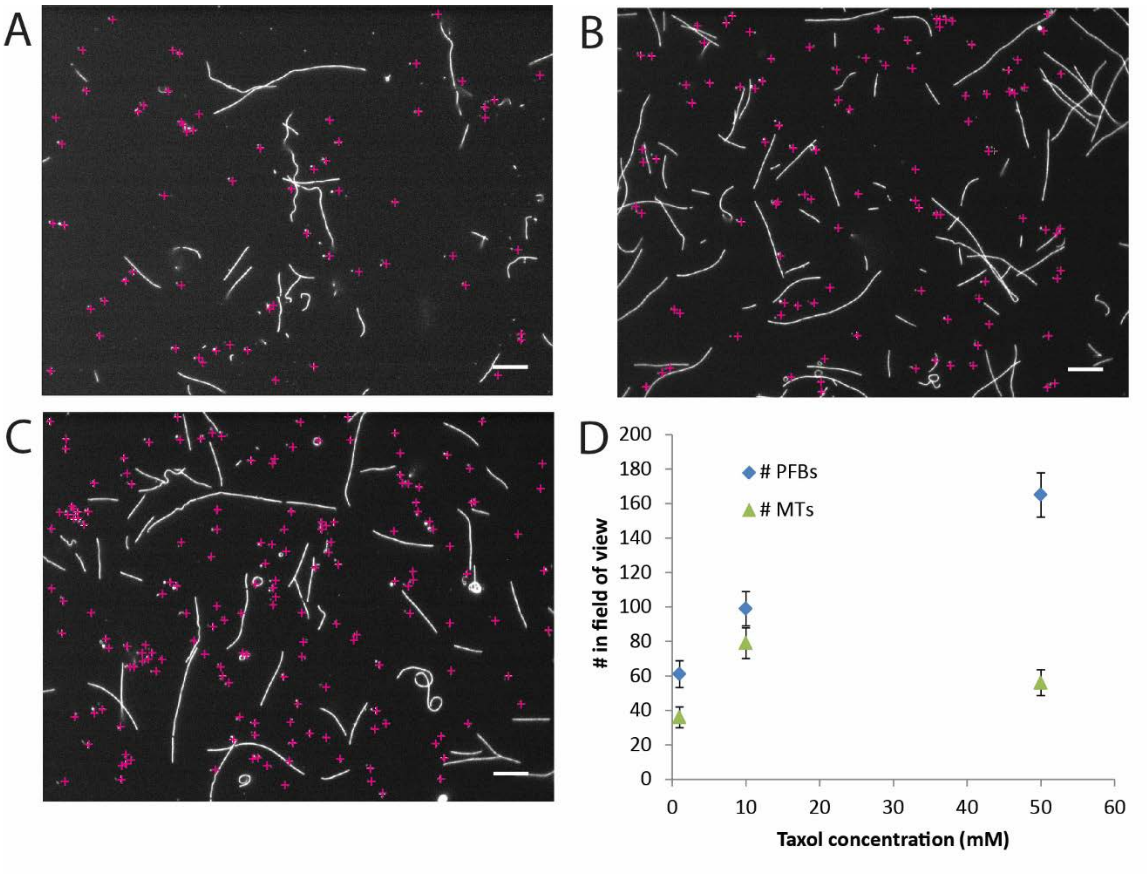

Figure S9. Fluorescence micrographs of experiments performed at 3 different paclitaxel concentrations of 1, 10, and 50 μM shown in (A), (B), and (C), respectively. PFBs are marked with magenta crosses. The surface density of GFP-kinesin in these experiments was 16500 μm^{-2}. Each assay was imaged 30 minutes after the final wash with motility solution. The same concentration of MTs was used in each experiment. The number of PFBs present is a function of the rate of PFB formation and the rate of PFB decay. Increased stability of PFBs is shown by having a higher concentration of PFBs present at 30 min with higher paclitaxel concentration. Scale bar is 10 μm. (D) Plot of number of PFBs and MTs in a field of view. Error bars are standard deviation.

Supplementary Figure 10

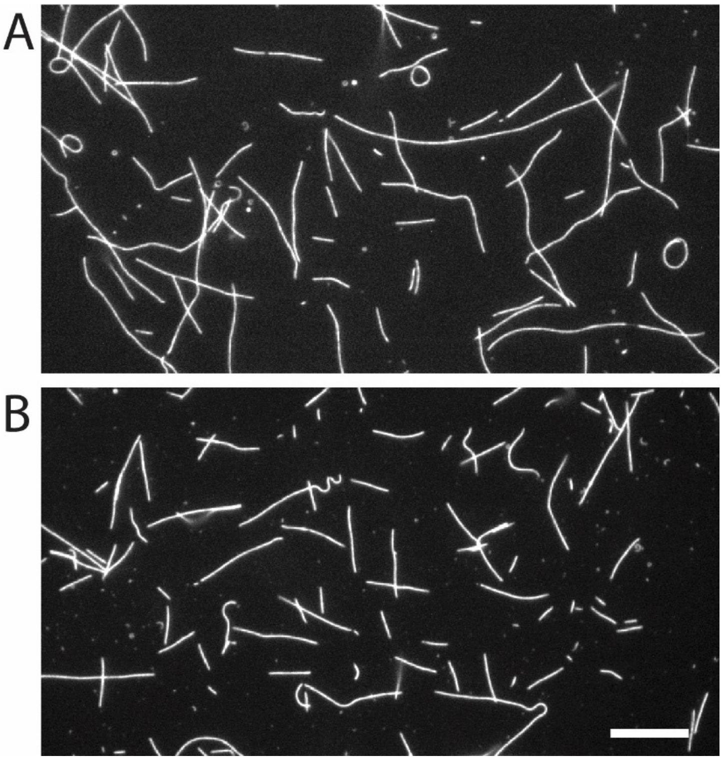

Figure S10. Fluorescence micrographs of experiments performed at with 20% and 50% TRITC-labelled tubulin MTs shown in (A) and (B), respectively. The surface density of GFP-kinesin in these experiments was 16500 μm^{-2}. Each assay was imaged 30 minutes after the final wash with motility solution. Scale bar is 10 μm.

Supplementary Figure 11

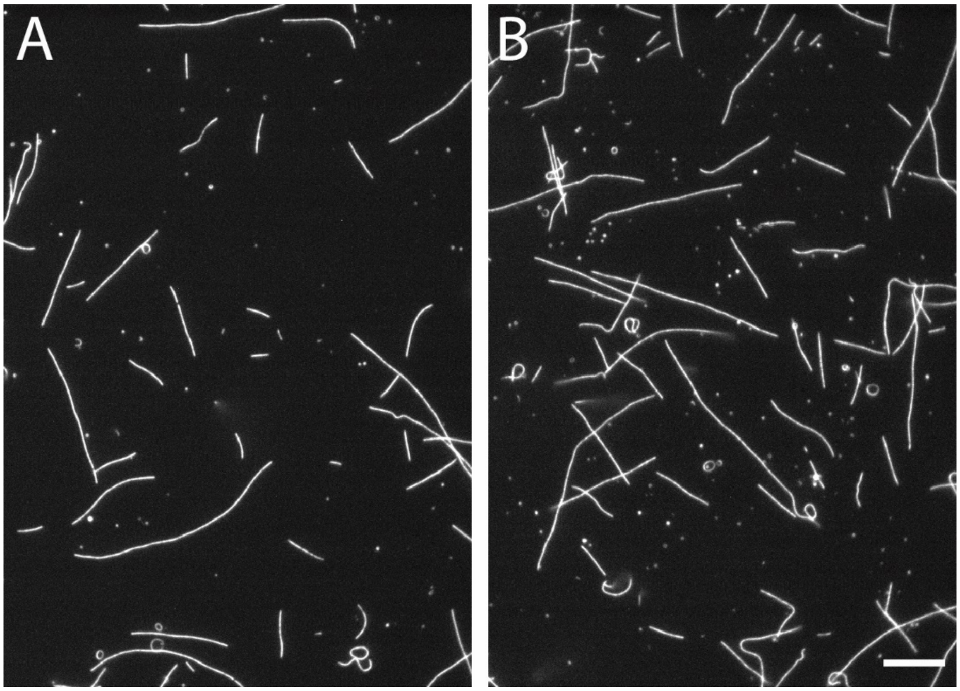

Figure S11. Fluorescence micrographs of experiments performed at with GTP of GMP-CPP in the MTs polymerization buffer shown in (A) and (B), respectively. The surface density of GFP-kinesin in these experiments was 16500 μm^{-2}. Each assay was imaged 30 minutes after the final wash with motility solution. The number of PFBs present is a function of the rate of PFB formation and the rate of PFB decay. Increased stability of PFBs is shown by having a higher concentration of PFBs present at 30 min for the GMP-CPP MTs. Scale bar is 10 μm.

Captions of Movies

Movie S1. Fluorescence movie of gliding assay of MTs transported by surface-bound GFP-kinesin showing MTs being split laterally into protofilament bundles.

Movie S2. Fluorescence movie showing the two MTs tips splitting into 2 fragments. The fragments have high curvature and are mobile.

Movie S3. Fluorescence movie of a fragment being pulled off of the side of a MT. Green arrow in first frame points to location of splitting event.

Movie S4. Fluorescence movie of a MT being led in a circular trajectory by a curved PFB at the leading end.
